# Timely Integration of specialist palliative care into oncology care for patients receiving radiotherapy for bone metastases: A study procotol for the TIPZO-RT Randomized Controlled Trial

**DOI:** 10.1371/journal.pone.0349792

**Published:** 2026-05-22

**Authors:** Anouk van Oss, Joanne M. van der Velden, Helena M. Verkooijen, Rebecca van Jaarsveld, Ginette M. Hesselmann, Evelien J.M. Kuip, Paulien G. Westhoff, Natasja J.H. Raijmakers, Yvette M. van der Linden, Roxanne Gal

**Affiliations:** 1 Center of Expertise in Palliative Care, Leiden University Medical Center, Leiden, The Netherlands; 2 Division of Imaging and Oncology, University Medical Center Utrecht, Utrecht University, Utrecht, The Netherlands; 3 Department of Radiotherapy, Division of Imaging and Oncology, University Medical Center Utrecht, Utrecht University, Utrecht, The Netherlands; 4 Imaging Cluster, Netherlands Cancer Institute, Antoni van Leeuwenhoek, Amsterdam, The Netherlands; 5 Division of Internal Medicine and Dermatology, University Medical Center Utrecht, Utrecht, The Netherlands; 6 Department of Medical Oncology and Department of Anaesthesiology, Pain and Palliative Medicine, Radboud University Medical Center, Nijmegen, The Netherlands; 7 Department of Radiotherapy, Radboud University Medical Center, Nijmegen, The Netherlands; 8 Netherlands Comprehensive Cancer Organisation (IKNL), Utrecht, The Netherlands; 9 Department of Radiotherapy, Leiden University Medical Center, Leiden, The Netherlands; PLOS: Public Library of Science, UNITED KINGDOM OF GREAT BRITAIN AND NORTHERN IRELAND

## Abstract

**Background:**

Patients living with advanced cancer often benefit from palliative care. Timely referral to specialist palliative care improves quality of life and reduces potentially inappropriate end-of-life care. Despite these benefits, specialist palliative care is frequently introduced late and inconsistently. This study evaluates whether systematically offering a consultation with the hospital palliative care consultation team (PCCT) to all patients referred for radiotherapy for symptomatic bone metastases improves satisfaction with care.

**Patients and Methods:**

The Timely Integration of Palliative Care in Oncology care for patients referred for palliative RadioTherapy (TIPZO-RT) trial follows the Trials within Cohorts design and is embedded within the PRospective Evaluation of interventional StudiEs on boNe meTastases (PRESENT+) cohort. Following cohort enrollment, 246 patients will be randomized (1:1) to either the intervention or control group. Patients in the intervention group are offered a PCCT consultation, which they may accept or refrain from. Patients in the control group are not informed about the trial and continue to receive usual care. After four weeks, patient satisfaction with care (affective behavior, EORTC Satisfaction with Cancer Care core questionnaire (EORTC PATSAT-C33)) will be compared between the groups. Secondary outcomes include symptom burden, quality of life, overall survival, and palliative care utilization. Additionally, in the intervention group, patients’ experiences with the consultation are evaluated.

**Discussion:**

Integrating palliative care into oncological care for patients with advanced cancer is essential to deliver comprehensive, patient-centered care that addresses physical, psychosocial and spiritual needs. This pragmatic study may provide evidence to support timely integration of specialist palliative care for all patients with bone metastases who may benefit from specialist palliative care. Using the Trials within Cohorts design, this study generates real-world evidence on the acceptance or need for a consultation with the PCCT, while minimizing disappointment or response bias, as patients in the control group are not informed.

**Trial registration:**

ClinicalTrials.gov ID NCT06805396. Registered on 25-03-2025.

## Introduction

During their disease trajectory, patients with advanced cancer and bone metastases are confronted with a variety of complaints in multiple dimensions including physical symptoms such as pain or side effects of oncological treatments, psychosocial distress and spiritual concerns. Palliative care aims to alleviate this suffering through early identification, comprehensive assessment, and treatment across physical, psychological, social and spiritual dimensions. The overall aim of palliative care is to improve the quality of life of patients with a life-limiting illness and also of their family caregivers, and can be delivered concurrently with oncological care [1,2].

Where healthcare professionals providing care for patients with a life-threatening disease are expected to provide basic (generalist) palliative care, palliative care specialists may be consulted in case of more complex situations, such as symptom burden on multiple dimensions [3,4]. Previous studies acknowledged the importance of timely referral to palliative care specialists. Randomized trials and meta-analysis investigating integrated palliative care in oncology for patients with advanced cancer have shown that it reduces symptom burden, improves quality of life, and helps prevent potentially inappropriate care at the end of life [5–10]. In a randomized controlled trial by Zimmermann et al., patients who received early palliative care (i.e., with an estimated survival of 6–24 months) reported higher satisfaction with care, whereas satisfaction deteriorated in the standard care (control) group [8].

Satisfaction with care is an important outcome for patients with advanced cancer, as they often require multiple clinical care services. It is hypothesized that palliative care enhances patient satisfaction with care by offering a person-centered holistic approach that addresses not only physical symptoms, but also psychological, social and spiritual needs [10,11]. In a previous randomized trial, patients who received early palliative care felt supported and guided in their disease trajectory and decision-making processes [8,12]. The hospital palliative care consultation team (PCCT) was particularly valued for taking the time to navigate patients through the healthcare system and help them prepare for the future, dicussing end-of-life care options consistent with individual patient preferences.

In the Netherlands, all hospitals providing oncological care are equipped with a PCCT where physicians, nurse practitioners and nurses, all with specialist palliative care training, may be consulted. According to SONCOS (Dutch Oncology Platform) guidelines, involvement of the PCCT should be considered in case of complex problems or upon patient request [3]. Despite clear evidence supporting the benefits of early integration of palliative care, referrals to palliative care specialists remain limited and may be impeded by clinician- and patient-related barriers [13,14]. In the Netherlands in 2024, only 15% of patients with cancer received a consultation with the hospital PCCT in their last year of life [15].

Clinician-related barriers include limited awareness or understanding of palliative care, the persistent misconception that palliative care is appropriate only at the very end of life, limited recognition of palliative care needs, time constraints, and competing clinical priorities [16–18]. Patient-related barriers include limited awareness of palliative care or associating it with end-of-life care, denial of the disease’s advanced stage and maintaining unrealistic expectations about life expectancy [17–19]. Furthermore, the process and timing of referral to specialist palliative care and who should be referred are poorly defined and varies across centers and specialties [20]. Multiple studies in the last decade aimed to identify criteria for timely referral of patients with advanced cancer to specialist palliative care [21,22]. However, referral of patients who meet these criteria requires efforts from clinicians to assess patients. Systematic referral of all patients to at least identify those patients in need of specialist palliative care may be more effective to timely integrate palliative care. A pilot study suggests that offering an introductory consultation with the PCCT at the time of radiotherapy referral for patients with bone metastases is both feasible and accepted. Patients valued the consultation and most did not perceive it as occurring too early in their disease trajectory [23].

### Aims

The primary objective of the TIPZO-RT trial is to evaluate the effect of systematically offering a consultation with the hospital PCCT on patient satisfaction with care four weeks after the initiation of radiotherapy in patients with advanced cancer undergoing treatment for bone metastases, compared to usual care (i.e., not systematically offering a consultation with the hospital PCCT).

Secondary objectives include 1) assessing the effect of systematic PCCT consultation on quality of life, symptom burden and overall survival, and 2) evaluating the utilization and perceived quality of specialist palliative care services in the intervention group.

## Patients and methods

### Participants

The TIPZO-RT trial follows the Trials within Cohorts (TwiCs) design and is embedded within the PRrospective Evaluation of interventional StudiEs on boNe meTastases (PRESENT+) cohort [24,25]. PRESENT+ includes patients with bone metastases referred to the Department of Radiotherapy [25]. The TIPZO-RT trial will be conducted within the Departments of Radiotherapy and the PCCTs of the University Medical Center (UMC) Utrecht, Radboudumc Nijmegen, and Leiden University Medical Center (LUMC) in the Netherlands. In addition, the PCCTs of the Meander Medical Center, Alrijne Hospital Leiden, and Groene Hart Hospital will participate in the study.

**Fig 1:**
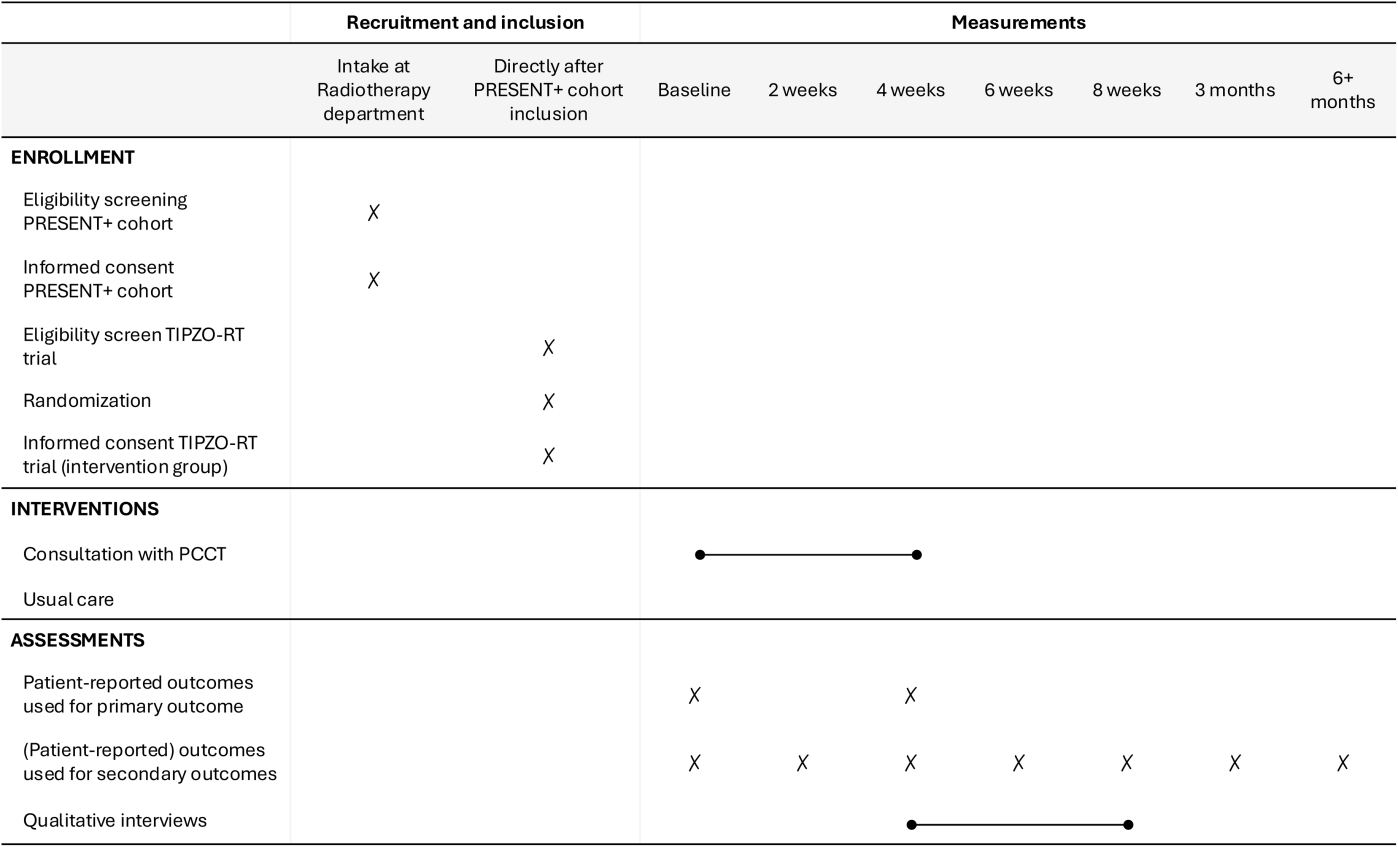
SPIRIT participant timeline: Schedule of enrollment, interventions, and assessments.

To be eligible to participate in the TIPZO-RT trial, patients must have provided broad informed consent for PRESENT + , including informed consent for use of clinical data for research, collection of patient-reported outcomes, and randomization into future trials (Fig 1). Patients will be excluded from participation if they 1) are unable to understand the study objectives, 2) lack the cognitive capacity to participate, or 3) previously had contact with the hospital PCCT.

### Study design

**Fig 2:**
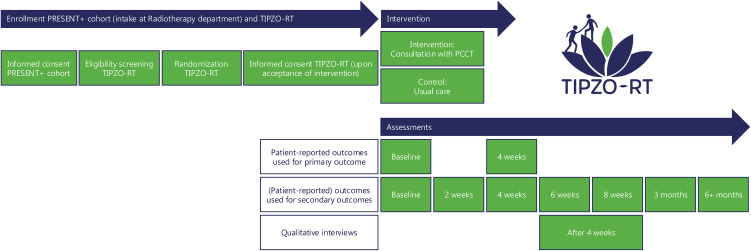
Participant timeline.

The TwiCs design offers a pragmatic approach to evaluate the effectiveness of interventions in real-world settings [24,26]. According to the TwiCs design, eligible patients will be randomized in a 1:1 ratio to the intervention or control group (Fig 2). Patients allocated to the intervention group will be contacted by a researcher by telephone, provided with study information using a standardised script and offered a consultation with the PCCT, which they may accept or refrain from. Patients in the control group will not be informed about the trial and receive usual care. Data collected within the cohort will be used to estimate intervention effects.

Randomization will be conducted using a computer-generated allocation sequence with random permuted blocks of sizes 4, 6 or 8 (Castor, Amsterdam, The Netherlands, www.castoredc.com). To ensure balance across sites, randomization will be stratified by participating center. Allocation concealment is maintained as the randomization code will only be released after the patient has been recruited into the trial.

Patients allocated to the intervention group will be informed about the intervention by the researcher, radiation oncologist or an authorized delegate, and will receive written information outlining the study procedures. Patients can accept or refrain from the intervention. If the patient needs additional time to consider participation, the researcher will contact the patient within a few days to determine whether the patient wishes to consult with a palliative care specialist of the PCCT. Patients who accept the intervention will be scheduled for an initial consultation with the palliative care specialist within two weeks after the start of radiotherapy. Informed consent will be obtained at the start of the PCCT consultation.

Blinding of researchers and clinicians to group assignment is not feasible. Patients in the control group remain blinded to group allocation and are unaware of the existence of the trial, consistent with the TwiCs design.

### Ethics and dissemination

This study falls outside the scope of the Dutch Medical Research Involving Human Subjects Act (Wet Medisch-wetenschappelijk Onderzoek met mensen, WMO) because the intervention is usual care and the randomization component is embedded within the PRESENT+ study, and therefore, formal approval of an accredited medical ethics committee is not required in the Netherlands [25,27]. However, in the UMC Utrecht, an independent quality check has been carried out to ensure compliance with legislation and regulations (regarding informed consent procedure, data management, privacy aspects and legal aspects). The main results of this study will be disseminated through publishing in peer-reviewed academic journals.

### Interventions

The palliative care consultation will be patient-centered, tailored to the wishes, needs and values of the patient, with family caregivers invited to be present and actively participate at the consultation. The palliative care consultant will conduct the consultation following their standard clinical procedures, guidelines, and practices. Before the consultation, all patients receive prepatory materials including a conversation aid and a site-specific PCCT leaflet. Furthermore, they are asked to complete a questionnaire (Utrecht Symptom Diary 4 Dimensions (USD-4D), Hospital Anxiety and Depression Scale (HADS) or Distress Thermometer), assessing the physical, psychological, social and spritiual dimensions, which serves as a guide for the consultation [28–31]. During the consultation, patients’ symptoms, wishes and needs across all four dimensions are discussed, followed by counseling and recommendations as appropriate, and referral to relevant specialists and/or follow-up of specialist palliative care if needed. The content of the consultation will be documented in the electronic health record and is accessible to both patients and their treating physician. In addition, the patients’ general practitioner will be notified of the consultation through a formal letter.

### Outcomes

The primary outcome is patient satisfaction with care, specifically focusing on the domain of affective behavior, measured four weeks after enrollment into PRESENT + . Satisfaction will be assessed using the European Organisation for Research and Treatment of Cancer (EORTC) Satisfaction with Cancer Care core questionnaire (PATSAT-C33) [32]. Patients will rate their experience on five-point Likert scales ranging from poor to excellent. The primary analysis will focus on the four-item subscale Affective Behavior. The PATSAT-C33 has been slightly adapted as participants will be asked to evaluate care provided by ‘healthcare providers in the hospital(s) in which they received oncological care’ instead of ‘nurses and radiotherapy technicians’.

Secondary outcomes will include patient-reported measures and overall survival:

- Pain (response), assessed at baseline, 2, 4, 6 and 8 weeks, and at 3 and 6 months, using the Brief Pain Inventory (BPI), which has been validated in patients with painful bone metastases [33,34]. The BPI consists of 11 items addressing pain perception and interference by pain. Responses are rated on a scale from 0 (no pain/interference) to 10 (maximum pain/interference).
- Symptom burden, including physical, psychological, social and spiritual symptoms, will be evaluated at 4 and 8 weeks, and at 3 and 6 months, using the Utrecht Symptom Diary – four dimensions (USD-4D), a 21-item instrument [28]. Items are scored on a 11-point scale ranging from 0 (no symptom) to 10 (worst intensity).
- Quality of life at baseline, 4 and 8 weeks, and at 3 and 6 months using the EORTC Quality of Life Questionnaire Core 15 Palliative Care (QLQ-C15-PAL), which evaluates physical and emotional functioning, and global quality of life [35].In addition, the EORTC QLQ Bone Metastases module (BM22) will be used to assess painful sites, pain characteristics, functional interference and psychosocial aspects [36]. Responses for each scale are rated on a four-point Likert scale (from ‘not at all’ to ‘very much’), summed and transformed to a 0–100 scale. Higher scores on functioning scales indicate better functioning, whereas higher score on symptom scales represent greater symptom burden. The global quality of life item is rated on a seven-point scale (from ‘very poor’ to ‘excellent’), with a higher transformed score reflecting a better overall quality of life.
- Additional patient satisfaction with care outcomes including information, coordination, and overall care, using the EORTC PATSAT-C33.
- Overall survival.

Secondary outcomes will also include palliative care utilization, assessed as follows:

- Within the intervention group, the number of patients who accept and complete the intervention (i.e., consultation with the PCCT) will be recorded, along with the documented reasons for refraining from the intervention.
- Specialist palliative care utilization during follow-up, defined as any contact with the hospital palliative care consultation team from randomization until death or up to two years post-randomization.
- Semi-structured in-depth interviews with patients who accepted the intervention will be conducted to obtain insight into experiences with the timing of the consultation and the consultation itself.

### Data collection, handling and monitoring

Consistent with the TwiCs design, data from the PRESENT+ cohort will be used for effect estimation. Patients who do not return a questionnaire within one week will receive a reminder. Patients who refrain or withdraw from the trial will continue to be followed within PRESENT + . If patients withdraw from PRESENT + , all data collected up to the point of withdrawal will be retained and included in the analyses.

De-identified data, including clinical data and data from the questionnaires, will be entered into the Castor data management system and is only accessible for investigators who are authorized to enter the system with their personal username and password. Based on the nature of the study interventions, a risk assessment has determined that risk-based monitoring is not necessary. Therefore, standard centralized oversight and routine data review are considered sufficient to ensure data quality and participant safety.

### Sample size

Based on internal observations (unpublished), we expect to enrol approximately 520 patients per year in PRESENT + . Currently, 79% of the patients participating in the PRESENT+ cohort provided consent to randomization into future intervention studies. Of those, approximately 40% are expected to meet the inclusion criteria for participation. Therefore, about 520 * 0.79 * 0.40 = 164 patients will be eligible every year for randomization in a 1:1 ratio to the intervention or control group. After 1.5 year of enrollment into the TIPZO-RT trial, we expect to have randomized 246 patients.

Of the patients offered a consultation with the PCCT, it is anticipated that 30% will refrain from the intervention. In a previous study, the mean score of patient satisfaction with care (interpersonal skills, EORTC IN-PATSAT32, which is most closely related to the primary outcome of this study (affective behavior, EORTC PATSAT-C33)) was 70.0 (SD = 21) among patients with metastatic solid tumors [37,38]. With a sample size of 246 patients, taking into account a refusal rate of 30% in the intervention group, 80% power, and a two-sided alpha of 0.05, the study is powered to detect a minimal difference of 7.6 points on patient satisfaction with care at four weeks follow-up (0.7 * 80.8 + 0.3 * 70.0).

### Statistical analysis

A detailed statistical analysis plan will be developed and published prior to data analysis. The primary analysis will compare the intervention group to the usual care control group in terms of patient satisfaction with care, specifically focusing on the subscale affective behavior. As some patients allocated to the intervention refrain from the intervention, the statistical analysis plan will outline how such non-adherence will be accounted for using the estimand framework [39,40]. Secondary and sensitivity analyses will be specified to address alternative clinical questions and analytical scenarios.

An intermediate analysis will be performed once 40% of the total sample has been randomized. This analysis will evaluate the proportion of patients that have completed the primary outcome measurement, as well as the refusal rate in the intervention group. If the refusal rate is higher than expected (i.e., > 10%), a sample size re-estimation may be considered to increase the sample size and maintain adequate power.

Approaches to handling non-adherence will be described in the statistical analysis plan and will be aligned with the estimand. The extent and patterns of missing data will be evaluated. If appropriate, multiple imputation methods will be used to address missing data under the assumption that data is missing at random (MAR). If the primary outcome data is determined to be missing not at random (MNAR), sensitivity analyses using best–worst and worst-best case scenarios will be performed to assess the robustness of the findings.

### Trial status

Patient recruitment started on April 28, 2025 and is expected to conclude in the first quarter of 2027. Data collection (of the primary outcome) will conclude four weeks after enrollment of the last patient. Study results are expected to be available in 2027 and will be submitted for publication.

## Discussion

Timely referral of patients with cancer who receive radiotherapy for symptomatic bone metastases to specialist palliative care remains a significant challenge, especially in this heterogeneous and clinical complex patient population requiring comprehensive, multidisciplinary care [17,41,42]. Due to the often complex symptom burden, including symptoms like pain, treatment-related side-effects, skeletal complications such as pathological fractures, neurological symptoms including spinal cord compression, alongside emotional, social and spiritual distress, and loss of mobility and independence, patients may require specialist palliative care. However, the integration of specialist palliative care into oncological care is not standard. Referrals are typically reactive, only when situations are complex, hindered by several barriers for both clinicians and patients.

Effective strategies are needed to ensure that all patients who may benefit from specialist palliative care are given the option to meet with a palliative care consultant. In order to achieve such, they should be adequately informed and appropriately referred. One potential strategy is the systematic referral of all patients, which ensures that every patient is made aware of the potential benefits of specialist palliative care and can make an informed decision about engaging with these services, now, or at a later stage during their disease trajectory. This approach empowers patients and family caregivers and enables timely access to specialized palliative care for those in need. However, there is a risk of overtreatment as not all patients require specialist palliative care and for some patients, generalist palliative care provided by the general practitioner, nurse specialists or treating physician may be more appropriate. Given the often limited availability of palliative care resources (e.g., personnel and costs), it remains important to deliver the right care at the right time and in the right setting.

A strategy for preselecting patients for specialist palliative care referral could be to refer only those patients who meet predefined criteria. A Delphi study involving clinicians in oncology and/or palliative care examined the use of predefined criteria to trigger systematic referral versus physician-based referral. The study reached consensus supporting combined use of systematic and physician-based referral [43]. The expert panel emphasized that systematic referral facilitates more frequent and timely referrals. In a randomized trial by Groenvold et al., patients with advanced cancer were first screened for palliative care needs using the EORTC QLQ-C30 questionnaire before being referred to palliative care [44,45]. Of the 1,146 patients screened, 621 patients (54%) did not meet criteria for palliative care needs based on symptom burden and functional impairment. Additionally, 124 of 464 patients eligible for randomization (27%) declined participation [45]. Palliative care needs are unlikely to be fully captured by questionnaires alone and may become apparement during the consultation.

Our study provides insight into the physical, psychosocial, and spiritual characteristics and symptoms of patients who accepted a consultation with the PCCT, reasons for refraining from the consultation, and whether patients perceived it as valuable or not.It will also be assessed whether involvement of a specialist PCCT was necessary or whether generalist palliative care would have been appropriate for most patients. These findings may inform future clinical practice in defining referral criteria and optimizing the timing to identify patients most likely to benefit from specialist PCCT. Given the limited availability of PCCT resources, such evidence is essential for developing effective triage strategies.

This study according to the TwiCs design aims to provide real-world evidence on the effect of systematic referral of patients with bone metastases to specialist palliative care [24,26,46]. The recruitment process mirrors clinical practice, thereby enhancing the external validity and generalizability of the findings. First, the pragmatic nature of the trial offers valuable insights into the uptake of specialist palliative care in patients with bone metastases, including the uptake of the intervention and the identification of patients who require specialist palliative care. Second, as control patients are not informed about the study, potential bias due to disappointment upon being assigned to the control group is avoided [47]. This approach mitigates risks of contamination (i.e., patients actively seeking the intervention) and dilution of the estimated treatment effect, as well as reduces the likelihood that patients discontinue participation due to dissatisfaction or selectively reporting less favorable outcomes based on knowledge of not receiving the promising intervention (response bias). Previous research indicated that most patients had positive or neutral reactions to being part of the control group without notification [47]. Third, by comparing the intervention to usual care, that is not systematically offering a consultation with the hospital PCCT at referral for palliative radiotherapy, this study evaluates the effectiveness of the intervention within current clinical practices. In usual care, clinicians may refer patients for specialist palliative care when deemed appropriate or when patients express a desire for referral. However, it is not common in current clinical practice that patients are referred, and if referred, it is often late in the disease trajectory [48].

## Conclusions

The pragmatic design of this TwiCs study has the potential to enhance timely consultation with hospital specialist palliative care consultants for all patients with cancer and bone metastases who are referred for radiotherapy and may benefit from integration of specialist palliative care into their care.
